# The Search for a Policy

**Published:** 1924-07

**Authors:** 


THE
### HOSPITAL and HEALTH
REVIEW

### THE SEARCH FOR A POLICY.

If there were any room for doubt that the most urgent need of the voluntary hospital system is a policy, it would be removed by the proceedings at various recent assemblies. The annual Conference of the British Hospitals Association, which has just been held with remarkable success in London, the recent first Conference, also eminently successful, of the Incorporated Association of Hospital Officers, the Conference between the Voluntary Hospitals Commission and the local Voluntary Hospitals Committee's held in the penultimate week of June were all directed, more or less prominently, to the imperative necessity of being prepared with a definite plan for the future of the voluntary institutions, for their co-ordination with each other and with similar institutions supported out of public funds, and their closer adaptation to needs which are not only growing but changing with almost disconcerting rapidity. All our old conceptions of the place of the voluntary hospital in the national life have to be cast aside and the field left clear for new ones; and the sooner this troublesome fact is recognised, the sooner will it be possible to mould these conceptions into shape and to start afresh. At present the situation is inchoate. Many ideas are in the air, but some are immature, others are conflicting, while authorities are not always agreed. Until this chaos of ideals is reduced to order and a clear policy deduced from them we shall not only be beating the air, but leaving open the gates of the citadel to the incursions of the enemy, who, in this case, are those who, for reasons that are often little better than political, are anxious to make an end of voluntaryism and replace it by a State-controlled and State-maintained system.

In these circumstances real importance attaches to two things which have just happened—the request of the Ministry of Health to the Voluntary Hospitals Commission to obtain a return of the existing accommodation in the institutions coming under that designation, and the plain-spoken paper on "The Future Relations of the Voluntary Hospitals to the Municipal Authorities," read by Dr. Kay Menzies to the British Hospitals Association, which will be found almost in its entirety elsewhere in our issue this month. The Commission must act through the Voluntary Hospitals Committees all over the country, who have now before them a detailed *Questionnaire* which before long will place the Commission, and through it the Government, in possession of an exact account of the number of beds in general and cottage hospitals, with an estimate in each case of the additional beds which their authorities deem to be necessary. This, and especially the latter, will be exceedingly useful information, and, indeed essential to a clear view of the subject. It should help materially towards the formation of a sound opinion upon the real scarcity of beds, a subject upon which, as Dr. Kay Menzies points out, there is a good deal of loose talk. To be told that the voluntary hospitals are gasping for beds, with a score of applicants for every one that becomes vacant, while ignoring the empty ones in Poor-law institutions is simply to create unreasonable and unfounded prejudice. When, therefore, Dr. Menzies says that the time has come for a "careful, comprehensive, and detailed survey and study of the situation," he will command general assent. What is needed is an exact statement of the hospital provision available from every source, voluntary, Poor Law, and Public Health—these matters can no longer be kept in water-tight compartments. The Conference of the British Hospitals Association formally, by resolution, adopted his language and instructed the Council to consider the best way of making the survey "and to give all the assistance in its power to any body that makes it."

We should be glad to see the Council come to the conclusion that the British Hospitals Association is of itself the right body to do this necessary work. Any "adequate and co-ordinated scheme for the prevention and treatment of disease throughout the whole country" ought to come, and will be expected to come, from the organisation which in addition to possessing an intimate knowledge of the position of each hospital, is well equipped, by its position as an interchange station, to form a sound general opinion upon the whole subject. By dealing broadly and comprehensively with a matter which closely affects not only the future of the voluntary system but the future health polity of the country, it could greatly strengthen its position and take a long step towards getting itself generally recognised as the unquestioned mouthpiece of the hospital world. It is of real importance that the new hospital policy should come from within and not be imposed from without. To formulate that policy before completing the inquiries that are now to be set on foot would be impossible. When it is formulated we shall have to take heed to the fact that we are legislating not for some need of the moment, such as a scarcity of beds, but for a long future in which hospitals of all kinds will fall into their place as part of a permanent and comprehensive national health system. At present we have no such system. We are working with a series of *disjecta membra* which, although they overlap and get into each other's way, fail to occupy the whole field, and leave an uncomfortable sense of something wanting. Yet the voluntary hospitals go far towards occupying it, and, whatever happens, they must be made the foundation of the new system as, for so many centuries, they have been the mainstay of the old. All the noblest emotions of mankind have been called into play for their creation and maintenance, and to abandon their voluntary character would be an affront to the best instincts of human nature, and a piece of incredible folly to boot.

